# The impact of *ACE2* and co‐factors on SARS‐CoV‐2 infection in colorectal cancer

**DOI:** 10.1002/ctm2.967

**Published:** 2022-07-22

**Authors:** Shuwei Li, Silu Chen, Qiuyuan Zhu, Shuai Ben, Fang Gao, Junyi Xin, Mulong Du, Haiyan Chu, Dongying Gu, Zhengdong Zhang, Meilin Wang

**Affiliations:** ^1^ Jiangsu Cancer Hospital, Jiangsu Institute of Cancer Research The Affiliated Cancer Hospital of Nanjing Medical University Nanjing China; ^2^ Department of Environmental Genomics, Jiangsu Key Laboratory of Cancer Biomarkers, Prevention and Treatment, Collaborative Innovation Center for Cancer Personalized Medicine Nanjing Medical University Nanjing China; ^3^ Department of Genetic Toxicology, The Key Laboratory of Modern Toxicology of Ministry of Education, Center for Global Health, School of Public Health Nanjing Medical University Nanjing China; ^4^ Department of Biostatistics, Center for Global Health, School of Public Health Nanjing Medical University Nanjing China; ^5^ Department of Oncology Nanjing First Hospital Nanjing Medical University Nanjing China

**Keywords:** ACE2, colorectal cancer, SARS‐CoV‐2, susceptibility

SARS‐CoV‐2 is the novel coronavirus leading to COVID‐19.^1^ Patients with cancer show a higher risk of infection with SARS‐CoV‐2 than patients without cancer.^2^, ^3^ *ACE2* ^4^ and two co‐factors, *TMPRSS2* and *FURIN*,^5^, ^6^ could be differentially expressed in various tissues involved in the susceptibility of cancer patients to SARS‐CoV‐2 infection. However, the functional role of these genes in colorectal cancer with COVID‐19 is not clear. This study is the first report to explore the expression pattern of *ACE2* and its co‐factors in colorectal cancer, as well as their effects on SARS‐CoV‐2 infection.

To assess the mRNA and protein levels of *ACE2* and two co‐factors in colorectal cancer, we performed RNA‐sequencing and proteomics analysis in both colorectal cancer tissues and adjacent normal tissues. *ACE2* was higher in colorectal tumour tissues than in normal tissues (Figure 1A–C), whereas *TMPRSS2* and *FURIN* were lower in tumour tissues compared to normal tissues (Figure 1D–H). The mRNA levels of *TMPRSS2* and *FURIN* gradually decreased with malignant progression in the course of normal, adenoma, and tumour (Figure 1I–L). However, no significant increase in *ACE2* mRNA was observed during malignant progression (Figure S1). The mRNA level of *ACE2* positively correlated with *TMPRRS2* and negatively correlated with *FURIN* (Figure S2). Stratification analysis showed that no significant differences in the age of onset, tumour site, or stage were related to the expression of these three genes (Figure S3).

**FIGURE 1:**
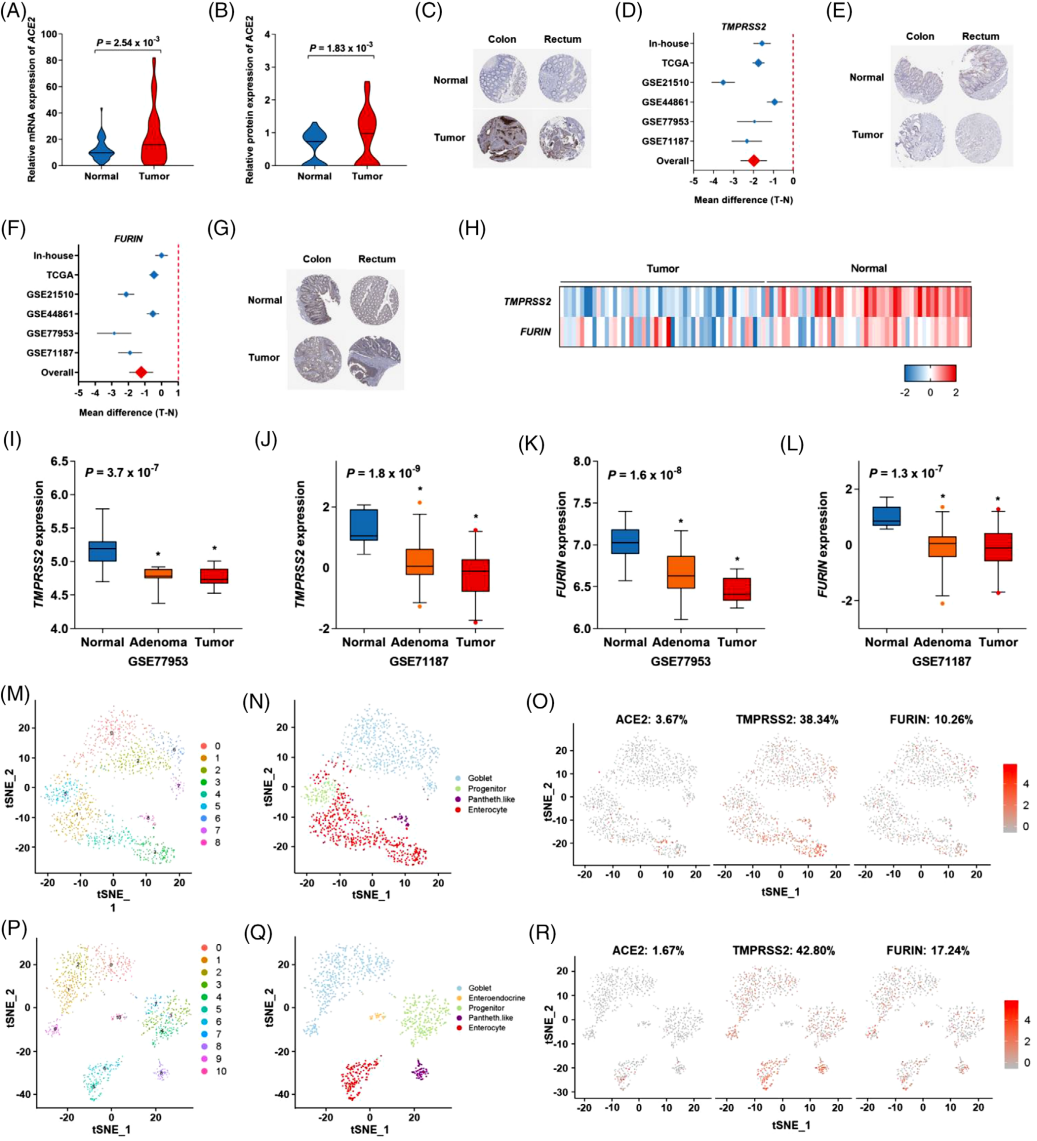
Differences in the expression of candidate genes between colorectal cancer tissues and normal tissues. (A) The mRNA expression of *ACE2* determined from RNA‐Seq. (B) The protein expression of *ACE2* determined by proteomics. (C) Representative immunohistochemical images of *ACE2* expression in colorectal tumour tissues from the HPA database. (D) Forest plots of *TMPRSS2* mRNA expression in six RNA‐Seq databases. (E) Representative immunohistochemical images of *TMPRSS2* in colorectal tumour tissues from the HPA database. (F) Forest plots of *FURIN* mRNA expression in six RNA‐Seq databases. (G) Representative immunohistochemical images of *FURIN* in colorectal tumour tissues from the HPA database. (H) Heat map of *TMPRSS2* and *FURIN* mRNA expression from the TCGA database. (I–L) The box plot of *TMPRSS2* and *FURIN* expression includes data from colorectal adenoma tissues from the GEO databases. (M) The tSNE plot displays the major cell clusters for the colon tissues from the GEO database (GSE125970). (N) The tSNE plot shows different cell types in the colon. (O) The tSNE plots show the expression of *ACE2*, *TMPRSS2*, and *FURIN* in the colon. (P) The tSNE plot displays the major cell clusters for the rectal tissues from the GEO database (GSE125970). (Q) The tSNE plot shows different cell types in the rectum. (R) The tSNE plots show the expression of *ACE2*, *TMPRSS2*, and *FURIN* in the rectum.

Based on single‐cell RNA‐sequencing profiling, *ACE2* was primarily expressed in enterocyte cells (Figures 1M–R and S4). *TMPRSS2* was primarily expressed in enterocytes and Paneth cells, and *FURIN* was expressed in all types of colorectal epithelial cells (Figure 1M–R). These results provide the co‐expression pattern of *ACE2* with its co‐factors in colorectal tissues.

Due to the low mRNA and protein expressions of *ACE2* in colon cells (Figure S5A and Table S1), we constructed colon cells with a stable overexpression of *ACE2* (Figure 2A–C). We transfected cells with the SARS‐CoV‐2 pseudovirus at an appropriate dilution (Figure 2D). Significantly increased luciferase activity and immunofluorescence were observed in *ACE2*‐positive colon cells compared to *ACE2*‐negative cells (Figure 2E,F). Because the level of *TMPRSS2* could influence SARS‐CoV‐2 infection, we further analysed the expression patterns of *TMPRSS2* and *ACE2*. We found a positive correlation between the levels of *ACE2* and *TMPRSS2* in multiple colorectal cell lines (Figure 2G). The mRNA and protein levels of *TMPRSS2* were increased with the increased mRNA and protein levels of *ACE2* (Figure S5B). Therefore, *ACE2* and *TMPRSS2* may play crucial roles in influencing SARS‐CoV‐2 infection in colorectal cells.

**FIGURE 2:**
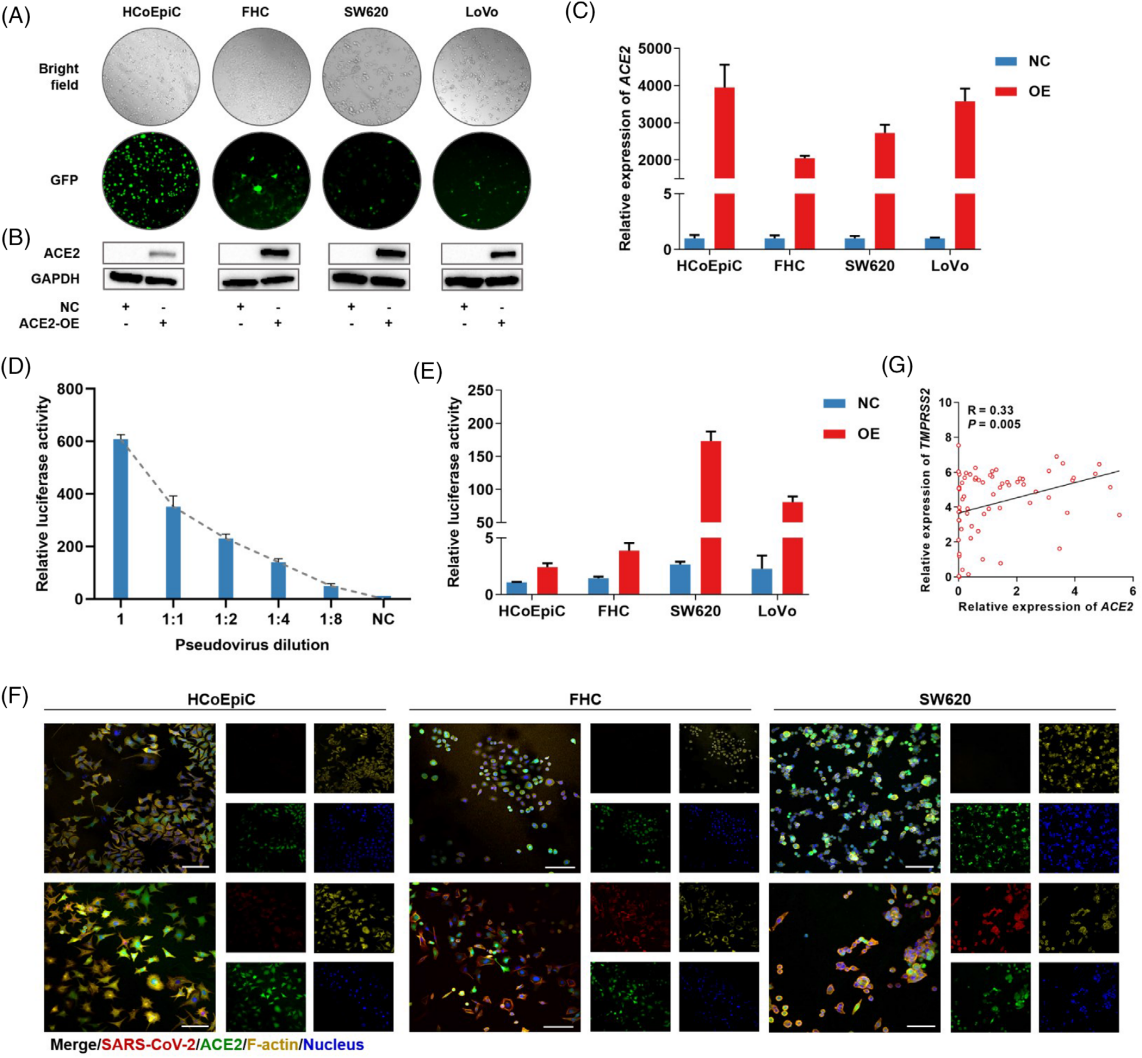
Different infection rates with SARS‐CoV‐2 between *ACE2*‐positive colon cell lines and negative control cells. (A) Transduction efficiency confirmed by GFP under a fluorescence microscope. Original magnification was 100×. (B and C) Stable overexpression of *ACE2* in the respective cell lines was measured using Western blotting (B) and RT‐PCR (C). (D) Quality control and appropriate delivery of SARS‐CoV‐2 pseudovirus were detected using luciferase reporter assays in SW620 cells. (E) Luciferase reporter assays were performed to measure SARS‐CoV‐2 infection in the respective cell lines with *ACE2* overexpression and control vectors. (F) Representative images of the immunofluorescence staining of SARS‐CoV‐2‐infected cells with stable overexpression of *ACE2* and control vectors. Cells were fixed and stained for the *ACE2* vector (GFP, green), SARS‐CoV‐2 (mCherry, red), F‐actin (phalloidin, yellow), and nucleus (DAPI, blue). Scale bars, 100 μm. (G) Linear correlation between *ACE2* and *TMPRSS2* in colorectal cancer cells based on the CCLE database.

Moreover, we compared the expression levels of *ACE2* and its co‐factors in different tissues. The ACE2 and FURIN protein expressions showed moderate‐to‐strong immunoreactivity, and the protein immunoreactivity of TMPRSS2 was low in most tumours (Figure S6). Notably, the *ACE2* and *TMPRSS2* mRNAs were expressed higher in colorectal tumour tissues than in many other tumours, such as lung, breast, and liver (Figure S7). These data indicate that even if *ACE2* and *TMPRSS2* are expressed at low levels in colorectal cancer, colorectal tissues may be particularly susceptible to SARS‐CoV‐2.

Previous studies demonstrated that *ACE2* correlated with immune infiltration.^7^, ^8^ The mRNA expressions of *ACE2* and its co‐factors were associated with immune infiltration in colorectal cancer (Figure S8, Table S2). Immune and stromal cells dominate the tumour microenvironment (TME) in cancer development. Therefore, we analysed the correlation between immunity‐related scores and the expression of *ACE2* and its co‐factors. The expression of *ACE2* and *TMPRSS2* negatively correlated with the immune score and stromal score (Figure 3A,B), and a positive correlation was detected between *FURIN* mRNA expression and these scores. Higher mRNA levels of *ACE2* and *TMPRSS2* were associated with lower overall activity of the anti‐cancer immune response (Figure 3C). These results suggested that higher mRNA expressions of *ACE2* and *TMPRSS2* influenced the TME in colorectal cancer.

**FIGURE 3:**
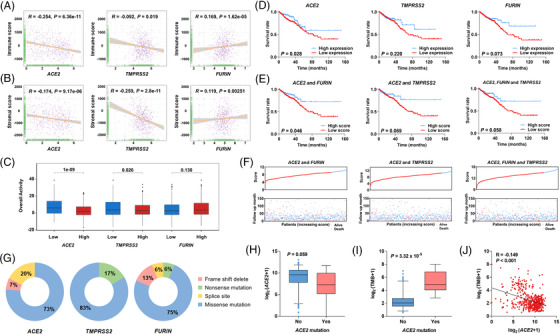
The association between the expression of candidate genes and the immune response, prognosis, and somatic mutation patterns in colorectal cancer tissues. (A and B) The correlation of the expression of three genes with immune score (A) and stromal score calculated by the ESTIMATE algorithm (B). (C) The association of the expression of three genes with the status of anti‐cancer immunity by the TIP algorithm. (D) Kaplan–Meier survival analysis of *ACE2* (left), *FURIN* (middle), and *TMPRSS2* (right) in colorectal cancer patients without details on whether they suffered from COVID‐19. (E) Patients were divided into low‐ and high‐expression groups according to the median expression of selected genes. Kaplan–Meier survival analysis of *ACE2* and *FURIN* (left), *ACE2* and *TMPRSS2* (middle), and all three genes (right). Patients were divided into high‐ and low‐score groups based on the median risk score under different combinations of selected genes. (F) The relationship between survival status, risk score rank (upper), and survival time (bottom). (G) Somatic mutation frequency of the three genes. (H) Box plot of *ACE2* expression in association with the status of *ACE2* mutations. (I) Box plot of tumour mutational burden (TMB) in association with the status of *ACE2* mutations. (J) Linear correlation between *ACE2* mRNA expression and TMB.

Furthermore, the low mRNA expression of *ACE2* was significantly associated with a poor survival time of patients, but no significant association was observed between the overall survival time and the levels of *TMPRSS2* or *FURIN* (Figure 3D). The overall survival time of patients in the higher level score group was longer than patients in the lower level score group for the combination of *ACE2* and *FURIN* but not for the combination of other groups (Figure 3E,F). These data suggested that *ACE2* may have dual functions in accelerating SARS‐CoV‐2 infection and prognosis in colorectal cancer.

We further explored the association of genetic variants in *ACE2* and its co‐factors with colorectal cancer susceptibility (Figures S9 and S10). However, no significant association was detected between candidate genetic variants and the susceptibility of colorectal cancer after false discovery rate correction (Table S3). Haplotypes with possible risk and the effect of each haplotype are shown in Table S4. The haplotype GCGGGGGTGA in *TMPRSS2* significantly decreased colorectal cancer risk compared to the most common haplotype GCGGGGGGGGGA (OR = .63, *p* = .031).

To identify somatic mutation patterns in *ACE2* and its co‐factors, we extracted mutational signatures of these genes in colorectal cancer. Missense mutations in *ACE2* (73.33%), *FURIN* (75.00%), and *TMPRSS2* (83.33%) were common in colorectal cancer tissues (Figure 3G and Table S5). Notably, we observed a significant increase in the mutation frequency of *ACE2* in the early‐onset colorectal cancer group compared to the late‐onset group (Table S6). We also detected a decreasing trend in *ACE2* expression in patients with *ACE2* mutations (Figure 3H). Tumour mutational burden (TMB) is an emerging biomarker for the immunotherapy response of cancers.^9^, ^10^ Therefore, we analysed the association of TMB with *ACE2* mutations and expression. TMB was higher in individuals with *ACE2* mutations compared to patients without *ACE2* mutations (Figure 3I) and was negatively associated with *ACE2* mRNA expression (Figure 3J).

In conclusion, we identified that *ACE2* was upregulated and positively correlated with *TMPRSS2* expression in colorectal cancer tissues. SARS‐CoV‐2 infection was significantly higher in *ACE2*‐positive colon cells. The mRNA expressions of *ACE2* and *TMPRSS2* were associated with the immune infiltration level of colorectal cancer. Our results suggest that *ACE2* and its co‐factors participate in the mechanisms underlying the association of colorectal cancer with COVID‐19 (Figure S11).

## CONFLICT OF INTEREST

The authors declare that there is no conflict of interest that could be perceived as prejudicing the impartiality of the research reported.

## Supporting information

Figure S1 The box plot of *ACE2* expression includes data from colorectal adenoma tissues from the GEO databases.
Figure S2 Linear correlation between ACE2 and its co‐factors.
Figure S3 Stratification analyses of the expression of *ACE2* and its co‐factors in subgroups.
Figure S4 Expression analysis of *ACE2* and *TMPRSS2*/*FURIN* using single‐cell RNA‐Seq in colorectal cancer cell clusters.
Figure S5 The expression patterns of ACE2 and its co‐factors in colorectal cancer cells.
Figure S6 Protein expression of *ACE2* and its co‐factors in different tumour tissues based on the HPA database.
Figure S7 mRNA expression of *ACE2* and its co‐factors in different tumour tissues based on the TCGA database.
Figure S8 The correlation of *ACE2* and co‐factor expression with immune infiltration in colorectal cancer tissues.
Figure S9 Summary of the design and workflow in the association study of genetic variants with colorectal risk.
Figure S10 The linkage disequilibrium plot exhibits the partition of pairwise variants in *ACE2*, *FURIN*, and *TMPRSS2* corresponding to the regional plot.
Figure S11 Schematic representation of SARS‐CoV‐2 infection in colorectal cancer patients.
Table S1 The protein expressions of ACE2, TMPRSS2, and FURIN in different colorectal cell lines.
Table S2 Correlation analysis between *ACE2*, *TMPRSS2*, and *FURIN* relates genes and markers of immune cells in TIMER.
Table S3 The association of genetic variants in *ACE2* and the co‐factors with colorectal cancer risk.
Table S4 Estimated frequency of haplotypes and the association with colorectal cancer risk.
Table S5 The mutation frequencies of *ACE2* and the co‐factors in colorectal cancer tissues by TCGA database.
Table S6 Stratification analyses for the association between mutation frequencies of three genes and different subgroups.
