# Supplementary material for: The impact of ACE2 and co‐factors on SARS‐CoV‐2 infection in colorectal cancer
Source: Clin Transl Med. 2022 Jul 22;12(7):e967. doi: 10.1002/ctm2.967 (PMC9305076; doi:10.1002/ctm2.967)
Supplement: Supplementary file 1 — Figure S1 The box plot of ACE2 expression includes data from colorectal adenoma tissues from the GEO databases. Figure S2 Linear correlation between ACE2 and its co‐factors. Figure S3 Stratification analyses of the expression of ACE2 and its co‐factors in subgroups. Figure S4 Expression analysis of ACE2 and TMPRSS2/FURIN using single‐cell RNA‐Seq in colorectal cancer cell clusters. Figure S5 The expression patterns of ACE2 and its co‐factors in colorectal cancer cells. Figure S6 Protein expression of ACE2 and its co‐factors in different tumour tissues based on the HPA database. Figure S7 mRNA expression of ACE2 and its co‐factors in different tumour tissues based on the TCGA database. Figure S8 The correlation of ACE2 and co‐factor expression with immune infiltration in colorectal cancer tissues. Figure S9 Summary of the design and workflow in the association study of genetic variants with colorectal risk. Figure S10 The linkage disequilibrium plot exhibits the partition of pairwise variants in ACE2, FURIN, and TMPRSS2 corresponding to the regional plot. Figure S11 Schematic representation of SARS‐CoV‐2 infection in colorectal cancer patients. Table S1 The protein expressions of ACE2, TMPRSS2, and FURIN in different colorectal cell lines. Table S2 Correlation analysis between ACE2, TMPRSS2, and FURIN relates genes and markers of immune cells in TIMER. Table S3 The association of genetic variants in ACE2 and the co‐factors with colorectal cancer risk. Table S4 Estimated frequency of haplotypes and the association with colorectal cancer risk. Table S5 The mutation frequencies of ACE2 and the co‐factors in colorectal cancer tissues by TCGA database. Table S6 Stratification analyses for the association between mutation frequencies of three genes and different subgroups. [file CTM2-12-0-s001.docx]

**The impact of *ACE2* and co-factors on SARS-CoV-2 infection in colorectal cancer**

**Supplementary materials and methods**

**Study populations and samples**

All of the participants in this study were unrelated Han Chinese individuals who provided written informed consent. A total of 52 colorectal cancer tumor tissue samples and paired adjacent normal tissue samples were collected from patients who were diagnosed and histopathologically confirmed tumors at the First Affiliated Hospital of Nanjing Medical University and Nanjing First Hospital beginning in September 2010, which were used for RNA-Sequencing (RNA-Seq). A total of 1,023 colorectal cancer patients and 1,306 cancer-free controls were enrolled in the case-control study, and the details were described in our previous study[^1^](#_ENREF_1). The institutional review board of Nanjing Medical University, China approved this study.

**RNA isolation and RNA sequencing (RNA-Seq)**

Total RNA was extracted using TRIzol Reagent (Invitrogen, CA, USA) according to the manufacturer’s protocol. The concentration and integrity of RNA were confirmed using a Nanodrop 2000 spectrometer (ThermoFisher Scientific) and an Agilent 2100 Bioanalyzer (Agilent, CA, USA). A total of 2 mg of RNA per sample was used as the input material for RNA sample preparation for library construction. Paired-end sequencing was performed using the Illumina HiSeq 2500 platform with 150 bp sequencing based on the Standard Illumina RNA-Seq protocol. Before the RNA-Seq data-filtering criteria were applied, the quality of the data was calculated using FastQC (version 0.11.7) software. The mapped reads were aligned to the GENCODE v19 genome assembly with HISAT2 and quantified with StringTie[^2^](#_ENREF_2). Cufflinks was used to quantify the relative expression of genes or transcripts and calculate the corresponding fragments per kilobase per million mapped reads (FPKM) values for each sample.

**Protein extraction and tryptic digestion for tandem mass tag (TMT) analysis**

The collected tissue samples were fixed in RNAlater stabilization solution (Invitrogen, Carlsbad, CA) and stored in a -80°C refrigerator for protein extraction. All of the samples were washed three times with phosphate-buffered saline (PBS) to remove blood and debris from the tissues. The samples were lysed in RIPA lysis buffer (Beyotime) supplemented with the protease inhibitor PMSF (Beyotime) on ice for 30 min. The lysates were sonicated for 10 min on ice and centrifuged at 20,000 × g for five min at 4°C to obtain clarified lysates. The protein concentration of the lysates was assessed using a bicinchoninic acid (BCA) protein quantitative kit (Sigma). Proteins were reduced for 1 h at 37°C using 5 mM dithiothreitol, followed by alkylation in 10 mM iodoacetamide at 25°C for 45 min in darkness. Quenching was performed at room temperature for 30 min with 30 mM cysteine. Trypsin digestion of the protein samples was performed, followed by desalting in C18 SepPak cartridges (Waters) and drying using a Speed-Vac apparatus. The desalted peptides were labelled with 10-plex TMT reagents according to the manufacturer’s instructions (Thermo Scientific). Peptides were fractionated using high-pH reversed-phase liquid chromatography (RPLC) to enhance the profiling depth of the proteome. Liquid chromatography-tandem mass spectrometry (LC-MS/MS) analysis was performed using a Thermo Scientific UltiMate 3000 RSLCnano system (ThermoFisher Scientific) coupled to a Q Exactive HF system (ThermoFisher Scientific), and proteome quantification analysis was performed using Proteome Discoverer at the protein level.

**Gene expression analysis in different tissues**

The mRNA expression profiles were downloaded from datasets of The Cancer Genome Atlas (TCGA) (http://cancergenome.nih.gov/) [^3^](#_ENREF_3), Gene Expression Omnibus (GEO) and Gene Expression Profiling Interactive Analysis (GEPIA) [^4^](#_ENREF_4). Immunohistochemical images and protein expression profiles were obtained from the Human Protein Atlas (HPA) database [^5^](#_ENREF_5). TCGA datasets were used to investigate the mRNA expression of selected genes (log2 transformed) between colorectal cancer and normal tissues. Normalized expectation-maximization read counts were available for 676 samples, including 625 colorectal cancer tumor tissues and 51 normal colorectal tissues. We also extracted the mRNA expression data from the GEO database (https://www.ncbi.nlm.nih.gov/geo/), including GSE21510[^6^](#_ENREF_6), GSE44861[^7^](#_ENREF_7), GSE77953[^8^](#_ENREF_8) and GSE71187[^9^](#_ENREF_9). The developed interactive GEPIA web server was used for tumor/normal tissue differential expression analyses in different types of cancers. The HPA database also provides immunohistochemical images and proteome profiles for different tissues and organs in the human body. We explored public transcriptome data based on single-cell RNA-Sequencing (scRNA-seq) for colon and rectal tissues. Briefly, the scRNA-seq data for colon and rectal epithelial cells were obtained from GSE125970^[10](#_ENREF_10)^ and the HPA database. Cell Ranger (version 3.1) was used to perform sample de-multiplexing, barcode processing and single-cell 5′ unique molecular identifier (UMI) counting. Cell barcodes were determined based on the distribution of UMI counts automatically. The following criteria were applied to each cell of the samples: gene number between 200 and 6,000; UMI count > 1,000; and mitochondrial gene percentage < 0.1. A filtered gene-barcode matrix of all samples was integrated using Seurat v.3 to remove batch effects across different donors. For the parameter settings, the first 50 dimensions of canonical correlation analysis (CCA) and principal-component analysis (PCA) were used.

Dimensionality reduction and clustering. The filtered gene-barcode matrix was first normalized using the “LogNormalize” method in Seurat v.3 with default parameters. The top 2,000 variable genes were identified using the ‘vst’ method in the Seurat Find Variable Features function. The variables “nCount_RNA” and “percent.mito” were regressed out in the scaling step, and PCA was performed using the top 2,000 variable genes. tSNE was performed on the top 50 principal components to visualize the cells. Graph-based clustering was performed on the PCA-reduced data for clustering analysis using Seurat v.3. The resolution was set to 1.2 to obtain a finer result.

**Cell transfection and SARS-CoV-2** **pseudovirus treatment experiments**

HEK-293T, FHC, SW620 and LoVo cells were obtained from the Type Culture Collection of the Chinese Academy of Science (Shanghai, China) and authenticated using short tandem repeat profiling at the time of purchase (2016–2021). HCoEpiC was obtained from the American Type Culture Collection in 2020. All cells were cultured in RPMI 1640 (Bionid) supplemented with 10% foetal bovine serum (Bionid) and 100 U/ml penicillin and streptomycin. For the stable overexpression of *ACE2*, cells were transfected with the GV358 lentivirus vector or negative control lentivirus (1×10^8^ TU/ml, MOI = 100), and stably transduced cells were selected with 2 µg/ml puromycin.

**Quantitative RT-PCR**

Total RNA was isolated using TRIzol reagent (Invitrogen, Carlsbad, CA, USA) and reverse-transcribed with PrimeScript RT Master Mix (Takara, Japan). Quantitative PCR was performed with SYBR Green reagents (Vazyme, China) and the following primers: *ACE2*, Primer-F 5′-AGAACCCTGGACCCTAGCAT-3′, Primer-R 5′-GCACATCCTCCTCCCCAAAA-3′; TMPRSS2, Primer-F 5′-ATTGCCGGCACTTGTGTTCA-3′; Primer-R 5′-ACAGTGTGCACCTCAAAGAC-3′; and *GAPDH*, Primer-F 5′-GAAATCCCATCACCATCTTCCAGG-3′, Primer-R 5′-GAGCCCCAGCCTTCTCCATG-3′.

**Western blotting analysis**

Protein concentrations were confirmed using a BCA protein quantitative kit (Sigma). Protein samples were separated in a 10% polyacrylamide gel and transferred to polyvinyl difluoride (PVDF) membranes (Bio-Rad, CA, USA). Antibodies against ACE2 (1:1000), TMPRSS2 (1:1000) and GAPDH (1:2000) were purchased from Cell Signaling Technology (USA), Abcam (Cambridge, UK) and Beyotime (Beyotime, China), respectively. Primary antibodies and goat anti-rabbit peroxidase-conjugated secondary antibodies (SA00001-2, Proteintech, China) were incubated with the membrane, and enhanced chemiluminescence (ECL) was used (Millipore, Billerica, USA) to assess the protein levels. GAPDH was used as an internal control.

**SARS-CoV-2 pseudovirus production assays**

The coding sequence of the VSV-G protein was replaced with the SARS-CoV-2 spike protein sequence in the lentiviral packaging system to produce the SARS-CoV-2 pseudovirus. The SARS-CoV-2 pseudovirus was co-transfected into HEK293T cells with the pLVX-mCherry-plasmid reporter gene using polyethyleneimine (PEI) (Polyscience, USA). After 48 h, we collected the cell supernatants containing the pseudovirus and titrated it to 10^5^ pfu/ml. The details were described previously [^11^](#_ENREF_11).

**SARS-CoV-2 pseudovirus infection and luciferase activity assays**

SARS-COV-2 pseudovirus was used to infect cells overexpressing ACE2 or negative controls at a mass ratio of 1:1,000 using Polybrene (Yeasen, China) according to the manufacturer's instructions. Twenty-four hours later, the pseudovirus medium was replaced with fresh medium, and the cells were incubated for an additional 48 h. The infection rate was determined using luciferase activity. Luciferase activity was measured using the Dual-Luciferase Reporter Assay System (Promega, USA). Relative luminescence signals were assessed by normalizing the luciferase signals to Renilla signals. Each cell line was used in three independent transfection experiments, and each experiment was performed in triplicate.

**Immunofluorescence staining**

FHC, HCoEpiC, SW620 and LoVo cells were washed twice with PBS and fixed in 4% (w/v) paraformaldehyde (P0098, Beyotime, China) for 20 min. The cells were permeabilized with 0.25% Triton X-100 (X100, Sigma-Aldrich, Germany) in PBS for 30 min at 4°C and washed three times with PBS. The cells were stained with DAPI (P0131, Beyotime, China) at room temperature for five min. The cells were washed three times with PBS and incubated with phalloidin (CA1670, Solarbio, China) for 20 min at room temperature. Images of fluorescent staining were obtained using laser scanning confocal microscopy (LSM880, Carl Zeiss, Germany) at room temperature.

**Calculation of immune and stromal scores using the ESTIMATE algorithm**

We used the Estimation of Stromal and Immune cells in Malignant Tumors using Expression data (ESTIMATE) algorithm in R (https://www.r-project.org/) to determine an immune score and stromal score for the TME of colorectal cancer tissues from the TCGA database. The Tracking Tumor Immunophenotype (TIP) algorithm was performed to analyze the overall activity related to the status of anti-cancer immunity.

**Tumor Immune Estimation Resource (TIMER) analysis**

TIMER is a web server for the systematic analysis of immune infiltrates in different cancers[^12^](#_ENREF_12)^,^ [^13^](#_ENREF_13). This comprehensive resource provides six major analytical modules for exploring the associations between immune infiltrates and different factors, such as gene expression, somatic mutation and clinical outcomes. We analyzed the association of the expression of selected genes with the abundance of infiltrating immune cell types. Expression scatter plots between selected genes were generated using the correlation module, Spearman’s correlation and the estimated statistical significance.

**Single-nucleotide polymorphism (SNP) selection and genotyping**

SNPs within genes and 2 kb upstream regions were screened from the 1000 Genomes Project (March 2012, Han Chinese in Beijing (CHB) data). The candidate SNPs were selected according to the following quality control criteria: (1) call rate ≥ 99%; (2) minor allele frequency (MAF) ≥ 10%; and (3) pairwise linkage disequilibrium analysis (*r*^2^ < 0.60). HaploReg v4.1 (http://archive.broadinstitute.org/mammals/haploreg/haploreg. php) and RegulomeDB (http://regulome.stanford.edu/index) were also used to predict the function of candidate SNPs. The processes of deriving genomic DNA and genotyping were described in our previous study[^1^](#_ENREF_1). Briefly, 500 μl peripheral blood samples from each subject were collected and preserved in ethylenediaminetetraacetic acid (EDTA) tubes. Genomic DNA was extracted from whole blood samples using the Qiagen Blood Kit (Qiagen).

**Somatic mutation and tumor mutation burden (TMB) analysis**

Whole-exome sequencing (WES) data, including somatic mutation information, were obtained from the TCGA database[^3^](#_ENREF_3). The somatic called variants were confirmed as the raw mutation count. TMB is an emerging biomarker of immunotherapy responses and was determined as the number of somatic mutations (including SNVs and InDels per megabase (MB) of genome examined) in the evaluated coding region. In detail, synonymous mutations were counted to reduce sampling noise. All base substitutions and indels in the coding region of targeted genes were initially counted before filtering, including the type of synonymous alterations. The total number of somatic mutations counted was divided by the coding region size in the targeted territory for TMB calculation.

**Statistical analysis**

All continuous data are presented as the means ± SD. A two-sided *P* value less than 0.05 was regarded as statistically significant. The χ^2^ test was used to evaluate differences in frequencies between the categorical variables in the groups. Statistical comparisons between two groups were performed using Student’s independent *t*-test or the Mann–Whitney *U* test, depending on the types of variables. Multiple comparisons were calculated using one-way analysis of variance (ANOVA). A linear regression model was used to evaluate the correlation between two factors. The survival curves of different groups were visualized using the Kaplan–Meier method. Power and sample size calculations were performed in Power and Sample Size (HyLown Consulting LLC; http://powerandsamplesize.com/). The association between candidate SNPs in selected genes and colorectal cancer risk was calculated using logistic regression with adjustment for sex and age in PLINK (version 1.9). Multiple testing correction was performed using the Benjamini-Hochberg false discovery rate (FDR). A two-sided *P*_FDR_ less than 0.10 was regarded as statistically significant in the genetic variant association analysis. Haplotype association analysis was performed by the haplo.stats R package. ORs are presented for comparisons of the risk haplotypes to the most frequent haplotype in each locus. Haplotypes with >1% frequency in the pool are presented.

**References**

1. Wang M, Gu D, Du M, Xu Z, Zhang S, Zhu L*, et al* Common genetic variation in ETV6 is associated with colorectal cancer susceptibility. *Nat Commun* **2016**; 7(11478).

2. Pertea M, Kim D, Pertea GM, Leek JT, Salzberg SL Transcript-level expression analysis of RNA-seq experiments with HISAT, StringTie and Ballgown. *Nat Protoc* **2016**; 11(9):1650-1667.

3. Weinstein JN, Collisson EA, Mills GB, Shaw KR, Ozenberger BA, Ellrott K*, et al* The Cancer Genome Atlas Pan-Cancer analysis project. *Nat Genet* **2013**; 45(10):1113-1120.

4. Tang Z, Li C, Kang B, Gao G, Zhang Z GEPIA: a web server for cancer and normal gene expression profiling and interactive analyses. *Nucleic Acids Res* **2017**; 45(W1):W98-W102.

5. Uhlén M, Fagerberg L, Hallström BM, Lindskog C, Oksvold P, Mardinoglu A*, et al* Proteomics. Tissue-based map of the human proteome. *Science* **2015**; 347(6220):1260419.

6. Tsukamoto S, Ishikawa T, Iida S, Ishiguro M, Mogushi K, Mizushima H*, et al* Clinical significance of osteoprotegerin expression in human colorectal cancer. *Clin Cancer Res* **2011**; 17(8):2444-2450.

7. Ryan BM, Zanetti KA, Robles AI, Schetter AJ, Goodman J, Hayes RB*, et al* Germline variation in NCF4, an innate immunity gene, is associated with an increased risk of colorectal cancer. *Int J Cancer* **2014**; 134(6):1399-1407.

8. Qu X, Sandmann T, Frierson H, Jr., Fu L, Fuentes E, Walter K*, et al* Integrated genomic analysis of colorectal cancer progression reveals activation of EGFR through demethylation of the EREG promoter. *Oncogene* **2016**; 35(50):6403-6415.

9. An N, Shi X, Zhang Y, Lv N, Feng L, Di X*, et al* Discovery of a Novel Immune Gene Signature with Profound Prognostic Value in Colorectal Cancer: A Model of Cooperativity Disorientation Created in the Process from Development to Cancer. *PLoS One* **2015**; 10(9):e0137171.

10. Wang Y, Song W, Wang J, Wang T, Xiong X, Qi Z*, et al* Single-cell transcriptome analysis reveals differential nutrient absorption functions in human intestine. *J Exp Med* **2020**; 217(2).

11. Liu X, Gao F, Gou L, Chen Y, Gu Y, Ao L*, et al* Neutralizing Antibodies Isolated by a site-directed Screening have Potent Protection on SARS-CoV-2 Infection. *bioRxiv* **2020**:2020.2005.2003.074914.

12. Li B, Severson E, Pignon JC, Zhao H, Li T, Novak J*, et al* Comprehensive analyses of tumor immunity: implications for cancer immunotherapy. *Genome Biol* **2016**; 17(1):174.

13. Li T, Fan J, Wang B, Traugh N, Chen Q, Liu JS*, et al* TIMER: A Web Server for Comprehensive Analysis of Tumor-Infiltrating Immune Cells. *Cancer Res* **2017**; 77(21):e108-e110.

**Figure S1.** The box plot of *ACE2* expression includes data from colorectal adenoma tissues from the GEO databases. (A) GSE71187. (B) GSE77953.


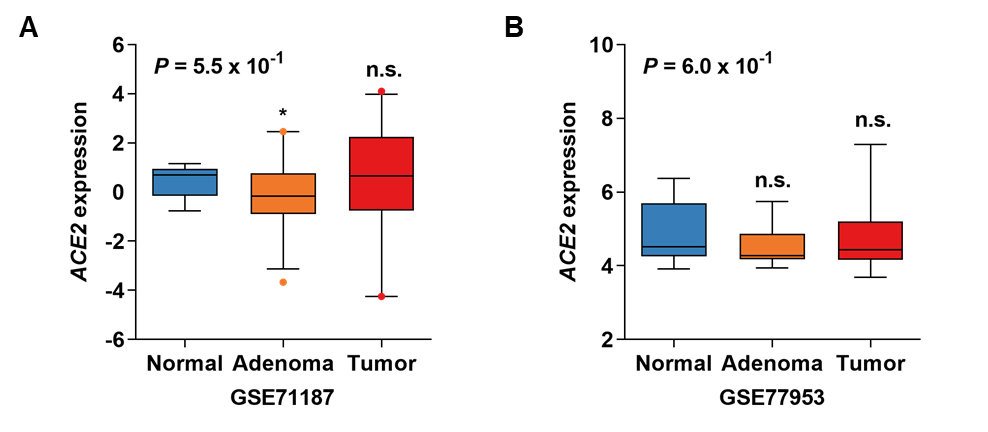


**Figure S2.** Linear correlation between *ACE2* and its co-factors. (A-C) The expression of *ACE2*, *TMPRSS2* and *FURIN* in colorectal cancer tissues based on the TCGA database.

**
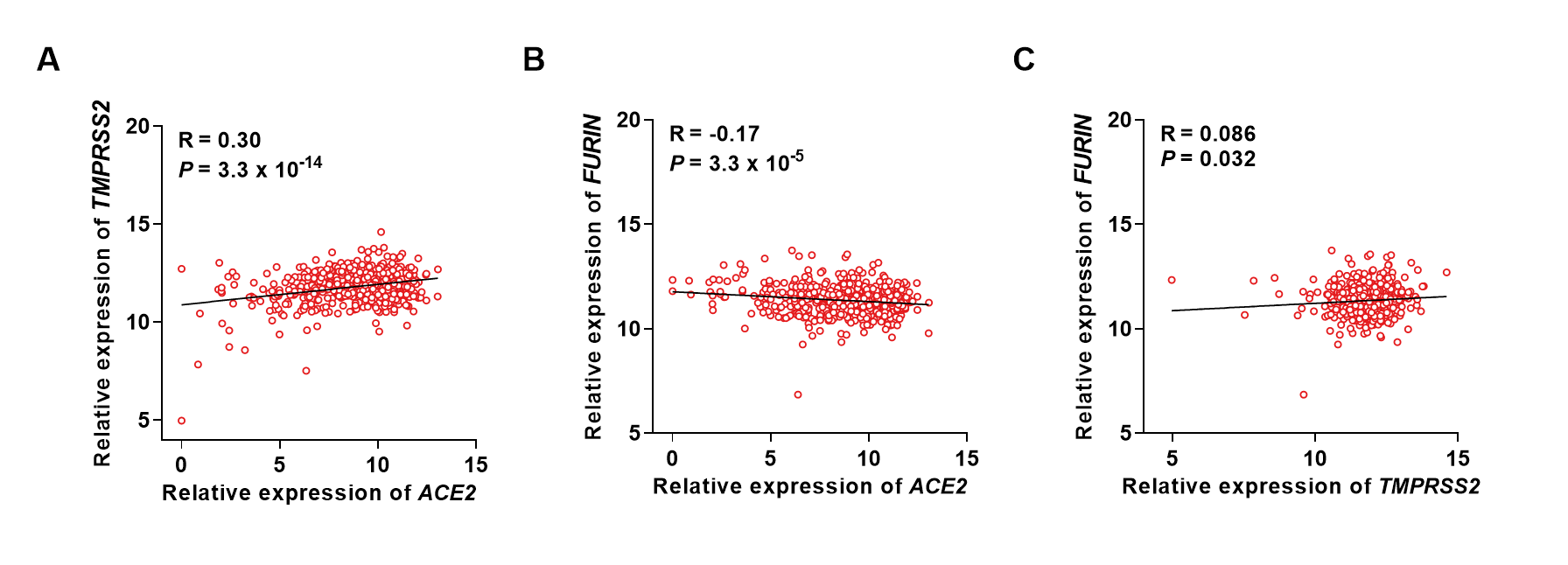
Figure S3.** Stratification analyses of the expression of *ACE2* and its co-factors in subgroups. (A-C) Linear correlation between the three genes and patient age. (D-F) Forest plots of *ACE2* (D), *TMPRSS2* (E) and *FURIN* (F) in clinical subgroups.

**
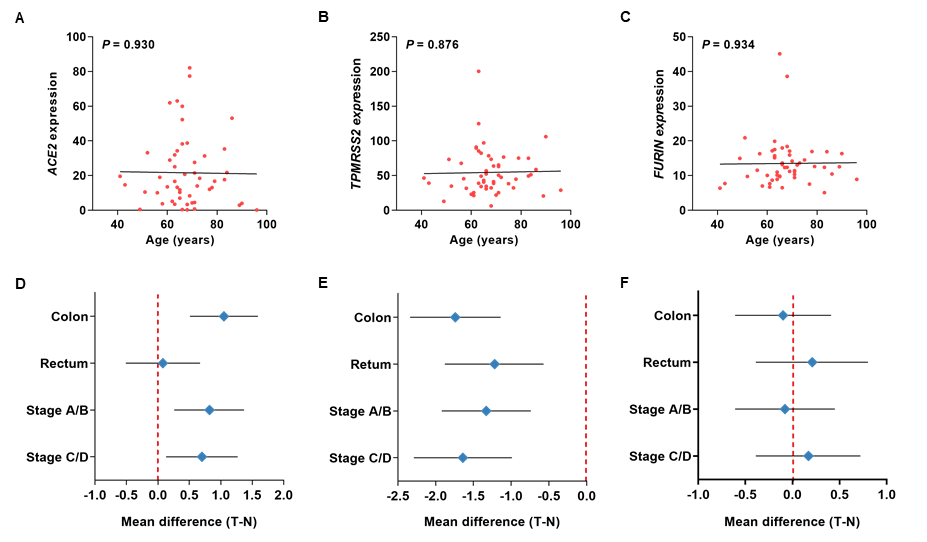
**

**Figure S4.** Expression analysis of *ACE2* and *TMPRSS2*/*FURIN* using single-cell RNA-Seq in colorectal cancer cell clusters. (A-B) The UMAP plot of *ACE2* RNA expression profile in colon (A) and rectum (B) tissues by single-cell RNA-Seq. (C-D) Bar chart of *ACE2* expression (pTPM) in each cell type cluster in colon (C) and rectum (D) tissues. Data were obtained from the HPA database.

**
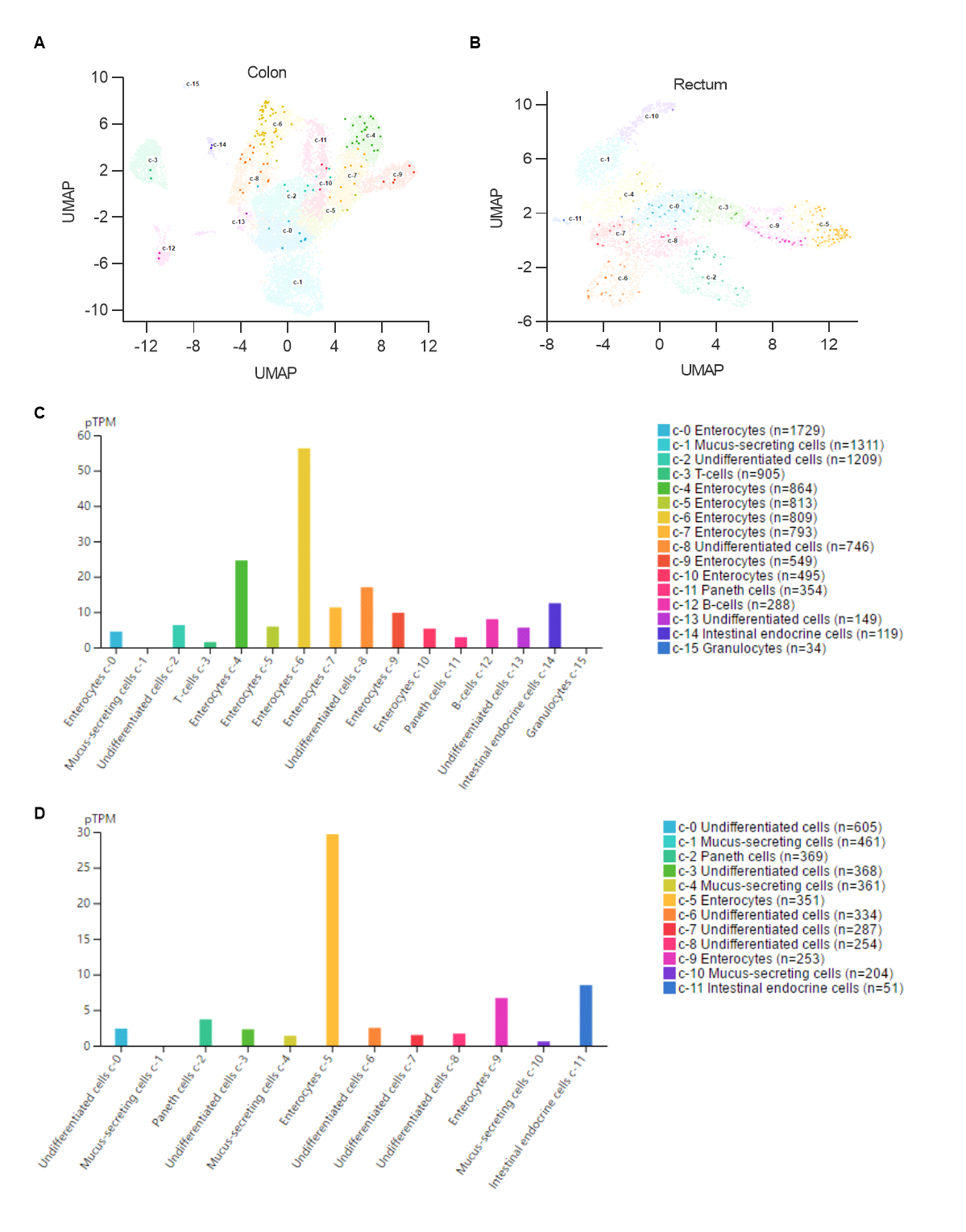
**

**Figure S5.** The expression patterns of *ACE2* and its co-factors in colorectal cancer cells. (A) The mRNA expression of *ACE2* and its co-factors in multiple colorectal cancer cell lines from the CCLE database. (B) The mRNA and protein levels of *TMPRSS2* in colorectal cell lines were detected using RT-PCR and Western blotting.

**
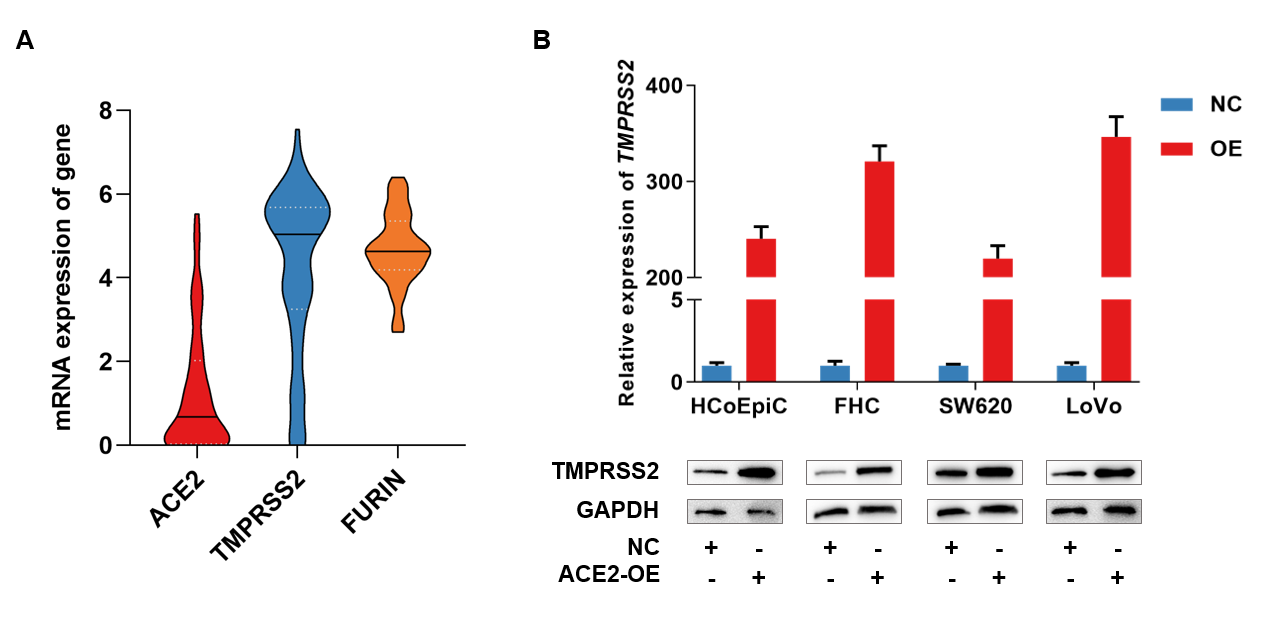
**

**Figure S6.** Protein expression of *ACE2* and its co-factors in different tumor tissues based on the HPA database. (A) *ACE2*. (B) *TMPRSS2*. (C) *FURIN*.

**
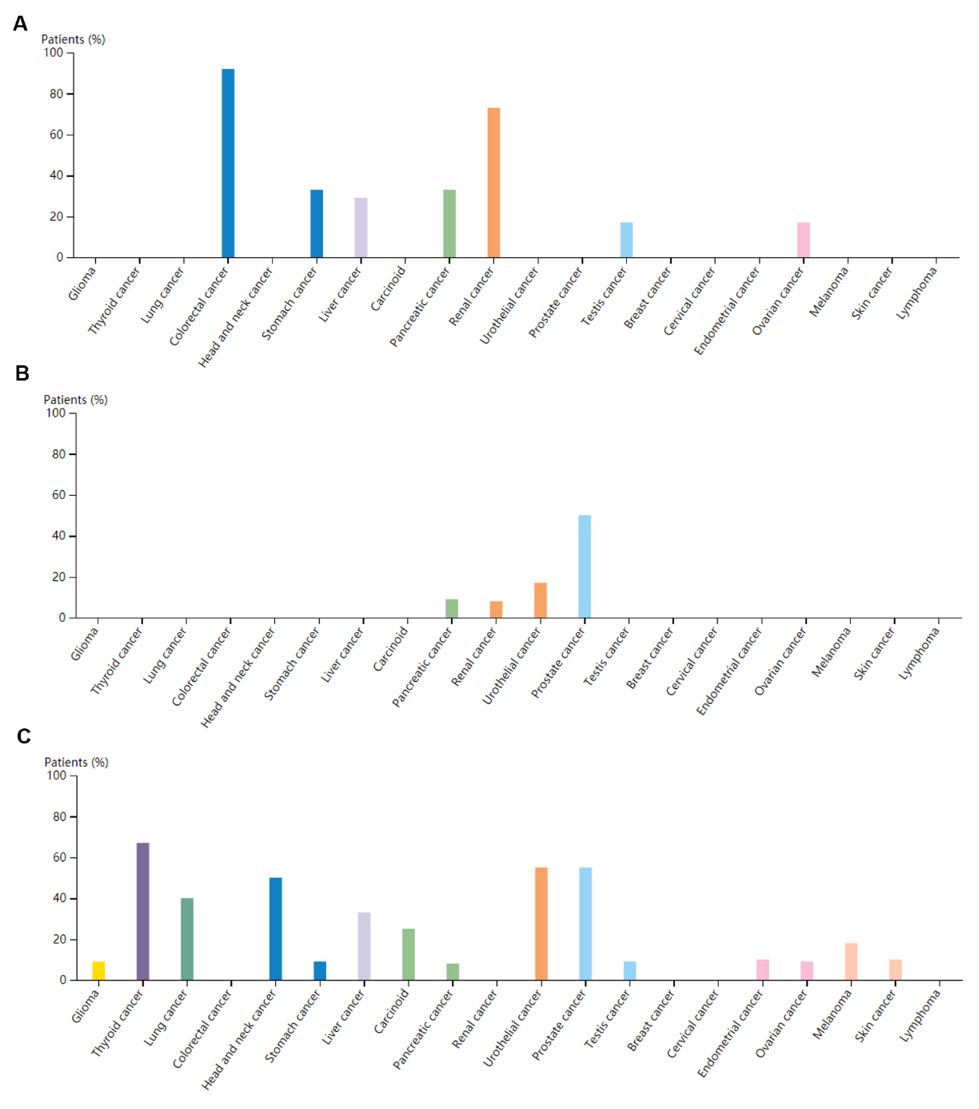
**

**Figure S7.** mRNA expression of *ACE2* and its co-factors in different tumor tissues based on the TCGA database. (A) *ACE2*. (B) *TMPRSS2*. (C) *FURIN*.

**
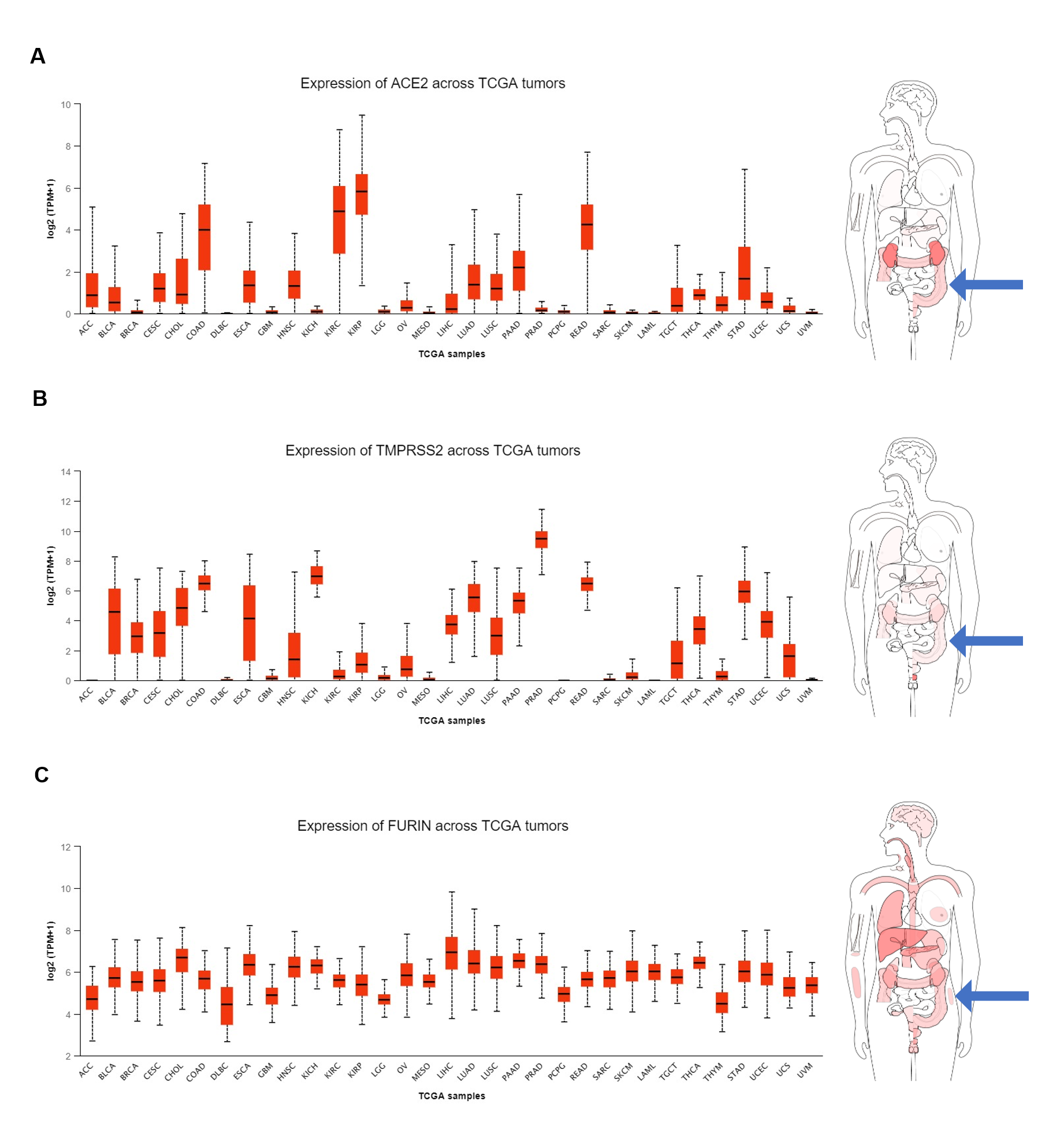
**

**Figure S8.** The correlation of *ACE2* and co-factor expression with immune infiltration in colorectal cancer tissues. The levels of immune infiltration in *ACE2* (top), *TMPRSS2* (centre), and *FURIN* (bottom) were obtained from the TIMER database.

**
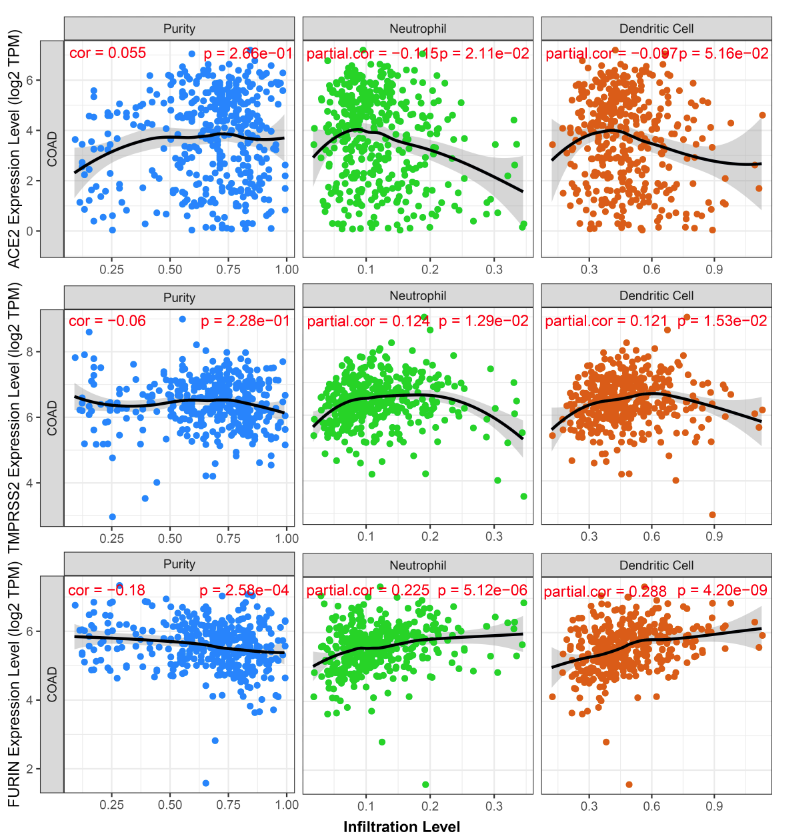
**

**Figure S9.** Summary of the design and workflow in the association study of genetic variants with colorectal risk. MAF, minor allele frequency. SNP, single nucleotide polymorphisms.


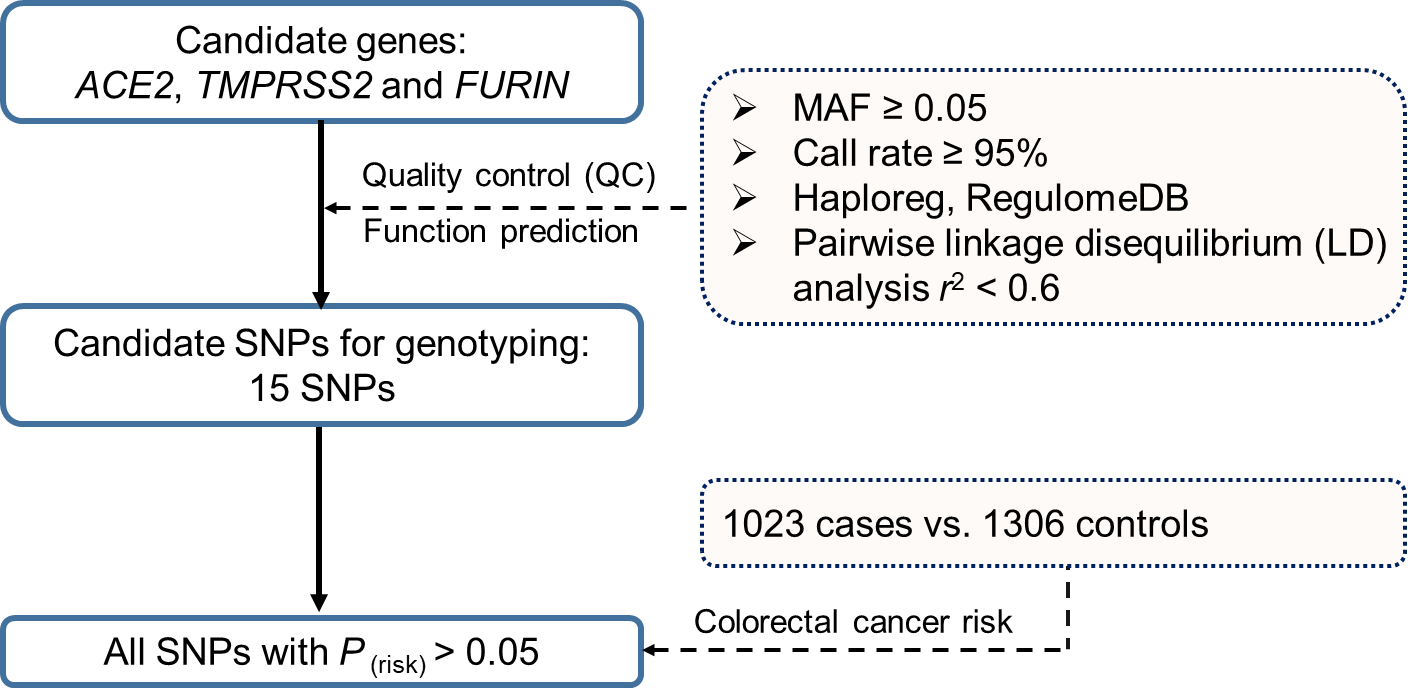


**Figure S10.** The linkage disequilibrium plot exhibits the partition of pairwise variants in *ACE2*, *FURIN*, and *TMPRSS2* corresponding to the regional plot. (A) *ACE2*. (B) *FURIN*. (C) *TMPRSS2*.


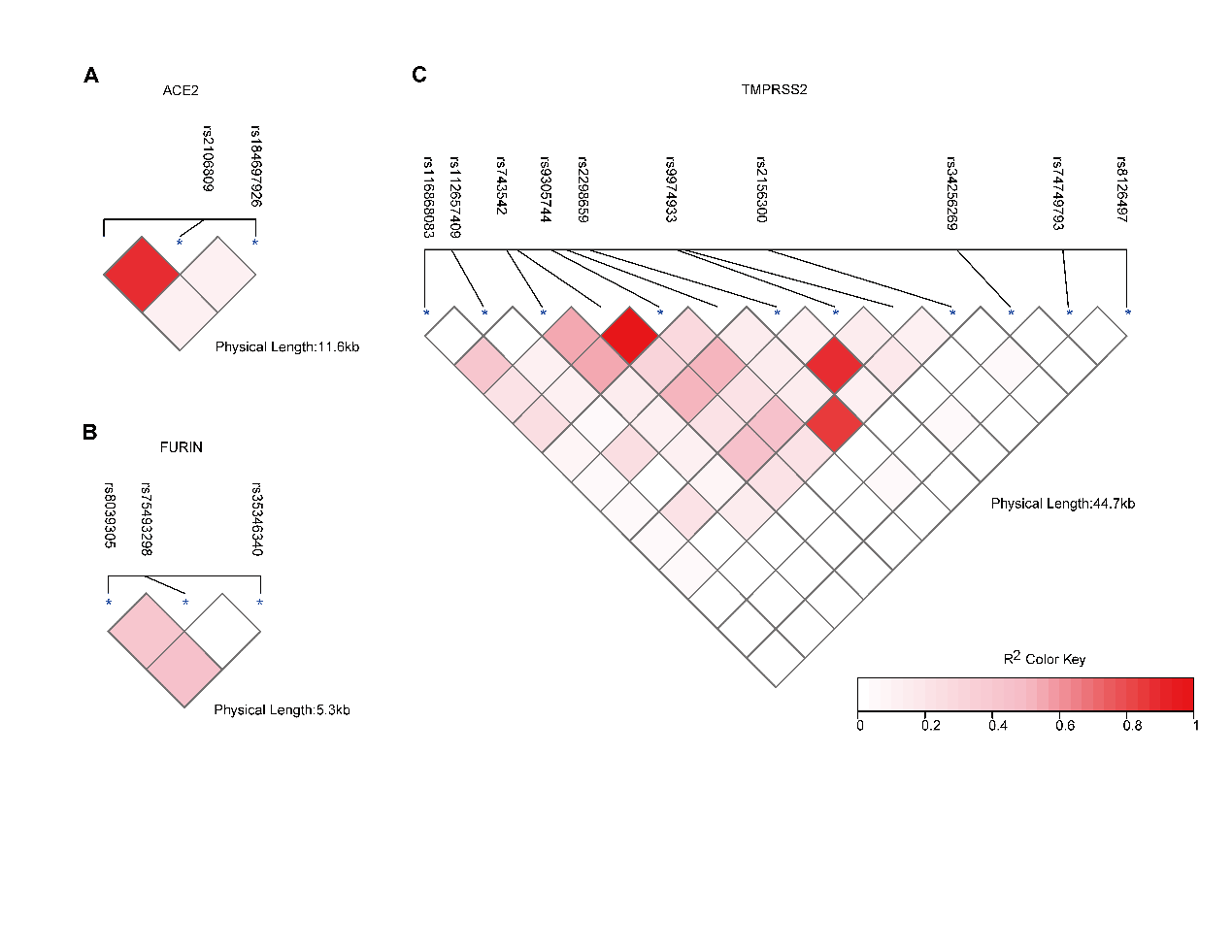


**Figure S11.** Schematic representation of SARS-CoV-2 infection in colorectal cancer patients. *ACE2* is upregulated in colorectal tumor tissues with the downregulation of *TMPRSS2* and *FURIN*. The expression of *ACE2* positively correlated with *TMPRSS2* in colorectal cancer tissues and cell lines. After the stable overexpression of *ACE2*, the infection rate of SARS-CoV-2 in colorectal cells was significantly higher than *ACE2*-negative colon cells. These three genes play specific roles in the abnormal immune response and infiltration levels in colorectal cancers and may predict the survival time of patients. The diagram was constructed using BioRender (https://biorender.com/).

**
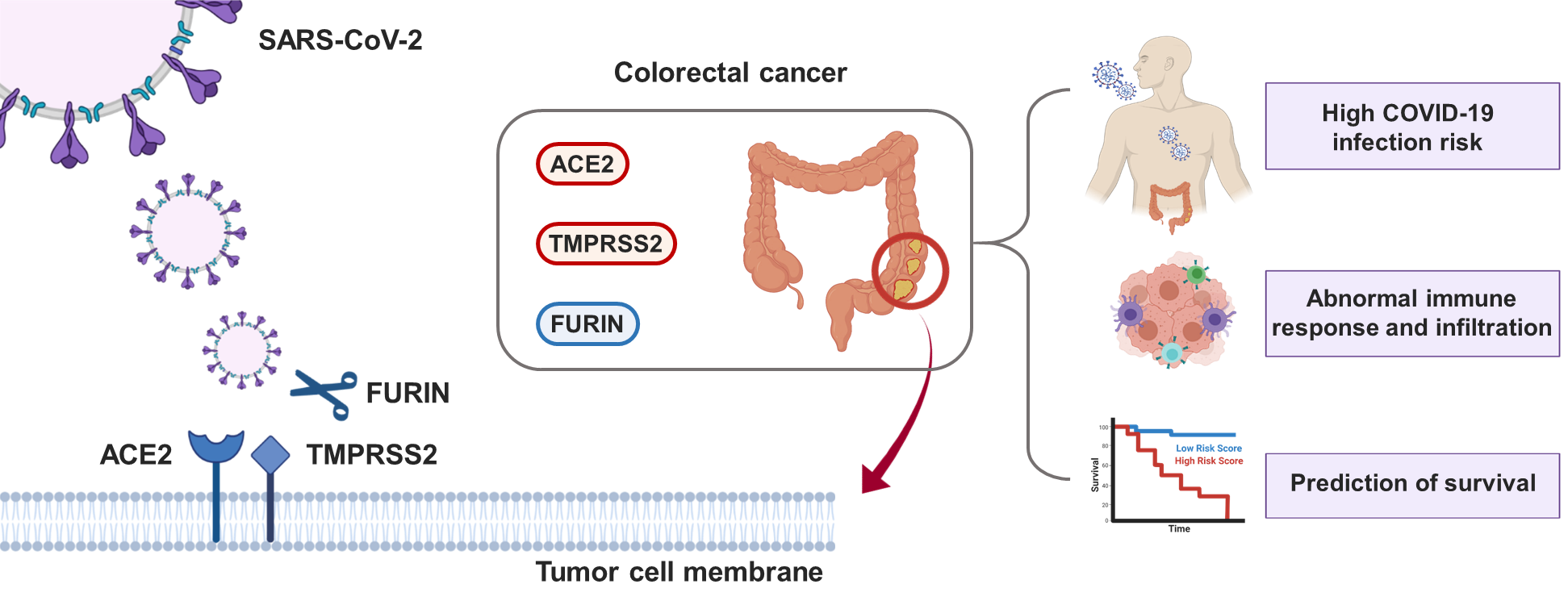
**

**Table S1. The protein expressions of ACE2, TMPRSS2 and FURIN in different colorectal cell lines.**

| Cell lines | Proteomics expression | | |
| --- | --- | --- | --- |
|  | ACE2 | TMPRSS2 | FURIN |
| LS513 | NA | 1.854881 | -0.35286 |
| SW1417 | NA | 1.904325 | 0.368688 |
| COLO678 | NA | -0.83345 | 1.398366 |
| SKCO1 | NA | 3.93036 | 0.590553 |
| HCC56 | NA | 3.233948 | NA |
| SNU61 | NA | 2.131793 | -0.88226 |
| SW620 | NA | 0.403895 | NA |
| SW948 | NA | 1.810978 | -0.82539 |
| SNUC1 | 2.506482 | 3.492605 | 0.099776 |
| SW403 | NA | 1.962608 | NA |
| HT55 | NA | 3.431568 | 1.225542 |
| MDST8 | NA | 0.925765 | NA |
| SW48 | NA | -1.04994 | NA |
| LS411N | NA | 0.787965 | -0.50025 |
| HCT15 | -0.41709 | -0.97585 | 0.47413 |
| COLO205 | NA | 2.914744 | 0.545287 |

NA, not detected.

**Table S2. Correlation analysis between *ACE2*, *TMPRSS2*, *FURIN* and relate genes and markers of immune cells in TIMER**

| **Description** | **Gene markers** | **Colon cancer** | | | | | |  | **Rectum cancer** | | | | | |
| --- | --- | --- | --- | --- | --- | --- | --- | --- | --- | --- | --- | --- | --- | --- |
|  |  | ***ACE2*** | | ***TMPRSS2*** | | ***FURIN*** | |  | ***ACE2*** | | ***TMPRSS2*** | | ***FURIN*** | |
|  |  | **Cor** | ***P*** | **Cor** | ***P*** | **Cor** | ***P*** |  | **Cor** | ***P*** | **Cor** | ***P*** | **Cor** | ***P*** |
| CD8+ T cell | CD8A | -0.229 | 5.12E-02 | -0.024 | 6.28E-01 | 0.160 | **1.22E-03** |  | -0.043 | 6.14E-01 | -0.059 | 4.92E-01 | 0.033 | 7.00E-01 |
|  | CD8B | -0.169 | 1.54E-01 | -0.116 | **1.91E-02** | 0.170 | **5.89E-04** |  | 0.102 | 2.30E-01 | -0.119 | 1.62E-01 | 0.103 | 2.29E-01 |
| T cell (general) | CD3D | -0.064 | 2.01E-01 | 0.037 | 4.56E-01 | 0.126 | **1.08E-02** |  | -0.013 | 8.75E-01 | -0.034 | 0.688 | -0.008 | 9.28E-01 |
|  | CD3E | -0.053 | 2.86E-01 | 0.060 | 2.26E-01 | 0.214 | **1.43E-05** |  | 0.039 | 6.49E-01 | 0.053 | 0.533 | 0.119 | 1.62E-01 |
|  | CD2 | -0.027 | 5.89E-01 | 0.044 | 3.77E-01 | 0.089 | 7.27E-02 |  | 0.078 | 3.62E-01 | 0.020 | 8.15E-01 | -0.011 | 9.00E-01 |
| B cell | CD19 | 0.118 | **1.70E-02** | 0.152 | **2.17E-03** | 0.157 | **1.55E-03** |  | 0.093 | 2.75E-01 | 0.032 | 7.09E-01 | 0.057 | 5.08E-01 |
|  | CD22 | 0.023 | 6.44E-01 | 0.101 | **4.18E-02** | 0.267 | **4.56E-08** |  | -0.004 | 9.66E-01 | 0.039 | 6.51E-01 | 0.170 | **4.52E-02** |
|  | CD70 | -0.154 | **1.84E-03** | -0.130 | **8.8E-03** | 0.079 | 1.11E-01 |  | 0.006 | 9.46E-01 | -0.194 | **2.23E-02** | 0.147 | **8.51E-02** |
|  | CD79A | 0.106 | **3.23E-02** | 0.145 | **3.32E-03** | 0.166 | **8.35E-04** |  | 0.052 | 5.46E-01 | 0.140 | 1.01E-01 | 0.051 | 5.52E-01 |
| Monocyte | CD86 | -0.150 | **2.38E-03** | -0.089 | 7.29E-02 | 0.102 | 4.06E-02 |  | 0.085 | 3.20E-01 | -0.014 | 8.67E-01 | 0.018 | 8.33E-01 |
|  | CD115 (CSF1R) | -0.106 | **3.23E-02** | -0.049 | 3.25E-01 | 0.529 | **9.87E-12** |  | -0.028 | 7.41E-01 | -0.036 | 6.71E-01 | 0.311 | **1.96E-04** |
| TAM | CCL2 | -0.056 | 2.61E-01 | -0.188 | **1.37E-04** | -0.001 | 9.86E-01 |  | -0.001 | 9.89E-01 | -0.162 | 5.6E-02 | 0.087 | 3.11E-01 |
|  | CD68 | -0.126 | **1.08E-02** | 0.000 | 9.99E-01 | 0.321 | **3.73E-11** |  | 0.070 | 4.14E-01 | 0.068 | 4.28E-01 | 0.210 | **1.29E-02** |
|  | IL10 | -0.064 | 1.95E-01 | -0.087 | 7.84E-02 | 0.078 | 1.16E-01 |  | -0.022 | 8.00E-01 | -0.098 | 2.49E-01 | 0.028 | 7.46E-01 |
| M1 Macrophage | INOS (NOS2) | 0.135 | **6.28E-03** | 0.386 | **7.42E-16** | 0.107 | **3.05E-02** |  | 0.051 | 5.53E-01 | 0.375 | 5.29E-06 | 0.218 | **9.80E-03** |
|  | IRF5 | 0.113 | **2.28E-02** | -0.089 | 7.45E-02 | 0.104 | **3.55E-02** |  | 0.211 | **1.26E-02** | -0.153 | 7.15E-02 | 0.207 | **1.43E-02** |
|  | COX2(PTGS2) | -0.087 | 7.99E-02 | 0.022 | 6.53E-01 | 0.107 | **3.13E-02** |  | 0.065 | 4.44E-01 | -0.009 | 9.2E-01 | 0.159 | 6.19E-02 |
| M2 Macrophage | CD163 | -0.180 | **2.71E-04** | -0.105 | **3.44E-02** | 0.226 | **4.08E-06** |  | -0.020 | 8.13E-01 | 0.015 | 8.63E-01 | 0.201 | **1.74E-02** |
|  | VSIG4 | -0.187 | **1.47E-04** | -0.206 | **2.86E-05** | 0.134 | **6.75E-03** |  | -0.017 | 8.38E-01 | -0.137 | 1.08E-01 | 0.025 | 7.66E-01 |
|  | MS4A4A | -0.170 | **5.92E-04** | -0.168 | **6.76E-04** | 0.078 | 1.17E-01 |  | -0.017 | 8.41E-01 | -0.064 | 4.55E-01 | 0.051 | 5.54E-01 |
|  | IRF4 | 0.092 | 6.40E-02 | 0.202 | **4.03E-05** | 0.163 | **9.73E-04** |  | 0.059 | 4.88E-01 | 0.132 | 1.21E-01 | 0.098 | 2.49E-01 |
| Neutrophils | CD66b (CEACAM8) | 0.303 | **4.32E-10** | 0.148 | **2.84E-03** | -0.152 | **2.11E-03** |  | 0.164 | 5.33E-02 | 0.087 | 3.07E-01 | 0.089 | 2.96E-01 |
|  | CD11b (ITGAM) | -0.209 | **2.25E-05** | -0.148 | **2.77E-03** | 0.256 | **1.73E-07** |  | -0.074 | 3.89E-01 | -0.024 | 7.81E-01 | 0.263 | 1.76E-03 |
|  | CCR7 | 0.093 | 6.04E-02 | 0.155 | **1.97E-03** | 0.302 | **5.56E-10** |  | 0.055 | 5.18E-01 | 0.068 | 4.28E-01 | 0.187 | **2.73E-02** |
| Natural killer cell | KIR2DL1 | -0.213 | **1.54E-05** | -0.152 | **2.19E-03** | 0.030 | 5.53E-01 |  | -0.081 | 3.44E-01 | -0.015 | 8.6E-01 | -0.096 | 2.59E-01 |
|  | KIR2DL3 | -0.274 | **2.03E-08** | -0.169 | **6.39E-04** | -0.012 | 8.04E-01 |  | -0.153 | 7.29E-02 | -0.126 | 1.41E-01 | -0.027 | 7.51E-01 |
|  | KIR2DL4 | -0.276 | **1.65E-08** | 0.035 | 4.86E-01 | 0.016 | **1.91E-02** |  | 0.020 | 8.14E-01 | -0.023 | 7.86E-01 | -0.013 | 8.81E-01 |
|  | KIR3DL1 | -0.248 | **4.23E-07** | -0.080 | 1.06E-01 | 0.032 | 5.18E-01 |  | -0.114 | 1.82E-01 | 0.063 | 4.6E-01 | -0.025 | 7.72E-01 |
|  | KIR3DL2 | -0.178 | **3.18E-04** | -0.049 | 3.2E-01 | 0.146 | **3.19E-03** |  | -0.134 | 1.16E-01 | -0.015 | 8.59E-01 | 0.131 | 1.23E-01 |
|  | KIR3DL3 | -0.117 | **1.88E-02** | 0.010 | 8.4E-01 | 0.062 | 2.14E-01 |  | -0.164 | 5.30E-02 | 0.007 | 9.34E-01 | -0.067 | 4.32E-01 |
|  | KIR2DS4 | -0.159 | **1.35E-03** | -0.089 | 7.39E-02 | 0.052 | 2.92E-01 |  | -0.087 | 3.07E-01 | -0.060 | 4.86E-01 | 0.122 | 1.51E-01 |
| Dendritic cell | HLA-DPB1 | -0.172 | **5.04E-04** | -0.094 | 5.95E-02 | 0.181 | **2.54E-04** |  | -0.103 | 2.28E-01 | -0.156 | 6.68E-02 | 0.131 | 1.26E-01 |
|  | HLA-DQB1 | -0.109 | **2.82E-02** | -0.076 | 1.26E-01 | 0.091 | 6.69E-02 |  | -0.006 | 9.43E-01 | -0.088 | 3.01E-01 | 0.047 | 5.85E-01 |
|  | HLA-DRA | -0.149 | **2.58E-03** | -0.035 | 4.79E-01 | 0.058 | 2.47E-01 |  | -0.074 | 3.90E-01 | -0.198 | **1.92E-02** | -0.083 | 3.33E-01 |
|  | HLA-DPA1 | -0.112 | **2.38E-02** | -0.040 | 4.16E-01 | 0.103 | **3.85E-02** |  | -0.036 | 6.70E-01 | -0.151 | 7.57E-02 | 0.004 | 9.64E-01 |
|  | BDCA-1(CD1C) | 0.198 | **5.91E-05** | 0.006 | 9.05E-01 | 0.087 | 7.98E-02 |  | -0.053 | 5.35E-01 | 0.002 | 9.84E-01 | 0.027 | 7.51E-01 |
|  | BDCA-4(NRP1) | -0.142 | **4.09E-03** | -0.066 | 1.85E-01 | 0.237 | **1.40E-06** |  | 0.012 | 8.88E-01 | 0.015 | 8.62E-01 | 0.160 | 6.07E-02 |
|  | CD11c (ITGAX) | -0.171 | **5.57E-04** | -0.049 | 3.62E-01 | 0.285 | **5.18E-09** |  | -0.069 | 4.20E-01 | -0.096 | 2.63E-01 | 0.257 | **2.23E-03** |
| Th1 | T-bet (TBX21) | -0.155 | **1.68E-03** | 0.004 | 9.4E-01 | 0.229 | **3.23E-06** |  | -0.021 | 8.04E-01 | -0.021 | 8.1E-01 | 0.147 | 8.44E-02 |
|  | STAT4 | -0.082 | 9.72E-02 | -0.011 | 8.3E-01 | 0.078 | 1.17E-01 |  | 0.012 | 8.89E-01 | -0.063 | 4.62E-01 | 0.000 | 9.96E-01 |
|  | STAT1 | -0.099 | **4.67E-02** | 0.081 | 1.03E-01 | 0.145 | **3.41E-03** |  | 0.124 | 1.47E-01 | 0.050 | 5.59E-01 | 0.032 | 7.05E-01 |
|  | IFN-γ (IFNG) | -0.130 | **8.97E-03** | -0.009 | 8.54E-01 | 0.034 | 4.95E-01 |  | -0.035 | 6.80E-01 | -0.005 | 9.52E-01 | -0.027 | 7.54E-01 |
|  | TNF-α (TNF) | 0.012 | 8.13E-01 | 0.079 | 1.14E-01 | 0.057 | 2.51E-01 |  | 0.140 | 1.01E-01 | -0.012 | 8.84E-01 | 0.225 | **7.77E-03** |
| Th2 | GATA3 | -0.011 | 8.30E-01 | 0.033 | 5.09E-01 | 0.225 | **1.88E-07** |  | -0.110 | 1.96E-01 | -0.028 | 7.47E-01 | 0.243 | **3.97E-03** |
|  | STAT6 | 0.098 | **4.81E-02** | 0.265 | **5.67E-08** | 0.386 | **7.20E-16** |  | -0.033 | 7.01E-01 | 0.222 | **8.59E-03** | 0.417 | **3.21E-07** |
|  | BATF | -0.105 | 3.51E-02 | -0.056 | 2.56E-01 | 0.056 | 2.64E-01 |  | -0.022 | 8.00E-01 | -0.032 | 7.12E-01 | 0.066 | 4.37E-01 |
|  | CD294 (GPR44) | 0.018 | 7.18E-01 | 0.039 | 4.32E-01 | 0.177 | **3.64E-04** |  | -0.057 | 5.03E-01 | 0.173 | **4.16E-02** | 0.154 | 6.96E-02 |
|  | IL13 | 0.065 | 1.89E-01 | -0.001 | 9.87E-01 | 0.091 | 6.83E-02 |  | -0.073 | 3.90E-01 | -0.109 | 2E-01 | -0.021 | 8.07E-01 |
| Tfh | BCL6 | -0.237 | **1.35E-06** | 0.055 | 2.66E-01 | 0.277 | **1.41E-08** |  | -0.173 | **4.19E-02** | -0.003 | 9.77E-01 | 0.228 | **6.86E-03** |
|  | IL21 | -0.053 | 2.88E-01 | -0.048 | 3.35E-01 | 0.023 | 6.42E-01 |  | 0.043 | 6.14E-01 | 0.050 | 5.6E-01 | -0.056 | 5.13E-01 |
|  | CD185 (CXCR5) | 0.041 | 4.07E-01 | 0.100 | **4.4E-02** | 0.279 | **1.02E-08** |  | 0.010 | 9.02E-01 | 0.109 | 2.03E-01 | 0.185 | **2.95E-02** |
|  | CD278 (ICOS) | 0.066 | 1.87E-01 | 0.068 | 1.69E-01 | 0.049 | 3.23E-01 |  | 0.182 | **3.16E-02** | 0.008 | 9.36E-01 | 0.030 | 7.29E-01 |
| Th17 | STAT3 | 0.120 | **1.59E-02** | 0.287 | **4.1E-09** | 0.272 | **2.42E-08** |  | 0.219 | **9.45E-03** | 0.392 | **1.87E-06** | 0.323 | **1.06E-04** |
|  | CD121a (IL1R1) | 0.038 | 4.44E-01 | 0.023 | 6.37E-01 | 0.238 | **1.11E-06** |  | 0.096 | 2.60E-01 | 0.115 | 1.79E-01 | 0.265 | **1.61E-03** |
|  | CD194 (CCR4) | 0.162 | **1.06E-03** | 0.143 | **3.97E-03** | 0.174 | **4.32E-04** |  | 0.160 | 5.94E-02 | 0.134 | 1.15E-01 | 0.100 | 2.43E-01 |
|  | CD196 (CCR6) | 0.292 | **1.93E-09** | 0.216 | **1.09E-05** | 0.044 | 3.76E-01 |  | 0.358 | **1.51E-05** | 0.317 | **1.45E-04** | 0.225 | **7.87E-03** |
|  | IL17 (IL17A) | 0.317 | **6.54E-11** | 0.236 | **1.49E-06** | -0.113 | **2.23E-02** |  | 0.077 | 3.65E-01 | 0.196 | **2.09E-02** | 0.100 | 2.41E-01 |
|  | IL21 | -0.053 | 2.88E-01 | -0.048 | 3.35E-01 | 0.023 | 6.42E-01 |  | 0.043 | 6.14E-01 | 0.050 | 5.6E-01 | -0.056 | 5.13E-01 |
|  | IL22 | 0.302 | **5.33E-10** | 0.226 | **4.42E-06** | -0.047 | 3.41E-01 |  | 0.097 | 2.56E-01 | 0.166 | 5.08E-02 | 0.153 | **7.25E-02** |
|  | IL23R | 0.313 | **1.15E-10** | 0.247 | **4.61E-07** | -0.040 | 4.23E-01 |  | 0.209 | **1.36E-02** | 0.435 | **8.72E-08** | 0.118 | 1.67E-01 |
| Treg | FOXP3 | 0.047 | 3.44E-01 | 0.058 | 2.41E-01 | 0.291 | **2.35E-09** |  | 0.115 | 1.78E-01 | -0.006 | 9.4E-01 | 0.269 | **1.37E-03** |
|  | CD25 (IL2RA) | -0.071 | 1.52E-01 | 0.038 | 4.42E-01 | 0.195 | **7.59E-05** |  | 0.041 | 6.29E-01 | 0.009 | 3.18E-01 | 0.152 | **7.40E-02** |
|  | CCR8 | 0.063 | 2.04E-01 | 0.063 | 2.02E-01 | 0.244 | **6.52E-07** |  | 0.145 | 8.83E-02 | 0.010 | 9.08E-01 | 0.126 | 1.38E-01 |
|  | STAT5B | 0.284 | **5.57E-09** | 0.135 | **6.6E-03** | 0.223 | **6.00E-06** |  | 0.207 | **1.46E-02** | 0.131 | 1.24E-01 | 0.242 | **4.04E-03** |
|  | TGFβ (TGFB1) | -0.181 | **2.49E-04** | -0.117 | **1.83E-02** | 0.346 | **7.06E-13** |  | 0.028 | 7.40E-01 | -0.153 | 7.24E-02 | 0.261 | **1.88E-03** |
| T cell exhaustion | PD-1 (PDCD1) | -0.161 | **1.16E-03** | 0.030 | 5.42E-01 | 0.256 | **1.78E-07** |  | -0.085 | 3.19E-01 | -0.046 | 5.93E-01 | 0.174 | **4.05E-02** |
|  | CTLA4 | -0.063 | 2.07E-01 | 0.003 | 9.45E-01 | 0.199 | **5.24E-05** |  | -0.035 | 6.84E-01 | -0.064 | 4.56E-01 | 0.100 | 2.42E-01 |
|  | LAG3 | -0.202 | **4.17E-05** | -0.011 | 8.27E-01 | 0.200 | **5.06E-05** |  | -0.074 | 3.88E-01 | -0.080 | 3.52E-01 | 0.144 | 9.15E-02 |
|  | TIM-3 (HAVCR2) | -0.194 | **8.64E-05** | -0.119 | **1.6E-02** | 0.103 | **3.81E-02** |  | 0.032 | 7.07E-01 | -0.129 | 1.32E-01 | 0.007 | 9.33E-01 |
|  | GZMB | 0.123 | **1.28E-02** | -0.025 | 6.2E-01 | -0.005 | 9.17E-01 |  | 0.226 | **7.49E-03** | -0.065 | 4.48E-01 | -0.036 | 6.74E-01 |

Cor, R value of Spearman’s correlation. TAM, tumor-correlated macrophage. Tfh, follicular helper T cell. Th, T helper cell. Treg, regulatory T cell

**Table S3. The association of genetic variants in *ACE2* and the co-factors with colorectal cancer risk.**

| Chr. | SNP | Position^a^ | RA | All sex | | |  | Male | | |  | Female | | |
| --- | --- | --- | --- | --- | --- | --- | --- | --- | --- | --- | --- | --- | --- | --- |
|  |  |  |  | OR^b^ | *P*^b^ | *P*^c^ |  | OR^b^ | *P*^b^ | *P*^c^ |  | OR^b^ | *P*^b^ | *P*^c^ |
| 15 | rs75493298 | 91423825 | T | 0.87 | 0.274 | 0.682 |  | 0.91 | 0.560 | 0.950 |  | 0.84 | 0.360 | 0.675 |
| 15 | rs8039305 | 91422543 | C | 1.03 | 0.698 | 0.873 |  | 0.95 | 0.660 | 0.950 |  | 1.14 | 0.322 | 0.675 |
| 15 | rs35346340 | 91427872 | C | 1.23 | 0.085 | 0.682 |  | 1.11 | 0.531 | 0.950 |  | 1.34 | 0.085 | 0.553 |
| 21 | rs34256269 | 42868876 | A | 0.80 | 0.102 | 0.682 |  | 0.72 | 0.102 | 0.950 |  | 0.91 | 0.599 | 0.803 |
| 21 | rs112657409 | 42836672 | T | 0.85 | 0.146 | 0.682 |  | 0.88 | 0.391 | 0.950 |  | 0.79 | 0.173 | 0.553 |
| 21 | rs116868083 | 42834988 | A | 0.89 | 0.200 | 0.682 |  | 0.99 | 0.906 | 0.981 |  | 0.78 | 0.076 | 0.553 |
| 21 | rs9305744 | 42842988 | A | 0.93 | 0.263 | 0.682 |  | 1.00 | 0.981 | 0.981 |  | 0.87 | 0.122 | 0.553 |
| 21 | rs2298659 | 42845374 | A | 0.95 | 0.499 | 0.745 |  | 0.97 | 0.737 | 0.950 |  | 0.94 | 0.558 | 0.803 |
| 21 | rs8126497 | 42879724 | A | 0.96 | 0.763 | 0.876 |  | 1.06 | 0.760 | 0.950 |  | 0.86 | 0.470 | 0.783 |
| 21 | rs2156300 | 42856756 | G | 0.96 | 0.546 | 0.745 |  | 0.92 | 0.362 | 0.950 |  | 1.00 | 0.975 | 0.975 |
| 21 | rs74749793 | 42875652 | T | 0.98 | 0.818 | 0.876 |  | 0.97 | 0.705 | 0.950 |  | 1.01 | 0.961 | 0.975 |
| 21 | rs743542 | 42840172 | A | 1.00 | 0.975 | 0.975 |  | 1.13 | 0.165 | 0.950 |  | 0.88 | 0.204 | 0.553 |
| 21 | rs9974933 | 42850977 | G | 1.06 | 0.387 | 0.682 |  | 1.01 | 0.949 | 0.981 |  | 1.14 | 0.221 | 0.553 |
| X | rs2106809 | 15618061 | A | 0.95 | 0.409 | 0.682 |  | 0.89 | 0.328 | 0.950 |  | 0.97 | 0.642 | 0.803 |
| X | rs184697926 | 15621988 | C | 1.10 | 0.381 | 0.682 |  | 1.24 | 0.270 | 0.950 |  | 1.01 | 0.960 | 0.975 |

Chr.: chromosome. RA: risk allele.

^a^Based on the NCBI database, build 37.

^b^*P* value of additive model in the logistic regression analysis.

^c^Multiple tests using false discovery rate levels

**Table S4. Estimated frequency of haplotypes and the association with colorectal cancer risk.**

| Gene | Haplotype^a^ |  | Estimated frequency | | |  | Effect | |
| --- | --- | --- | --- | --- | --- | --- | --- | --- |
|  |  |  | Pool  (N = 2,329) | Case  (N = 1,023) | Control  (N = 1,306) |  | OR (95% CI)^b^ | *P*^b^ |
| *FURIN* | GTG |  | 85.86% | 85.74% | 85.95% |  | 1.00 (Reference) |  |
|  | GCC |  | 7.59% | 8.42% | 6.93% |  | 1.21 (0.97-1.51) | 0.092 |
|  | TCG |  | 6.38% | 5.78% | 6.86% |  | 0.87 (0.68-1.12) | 0.290 |
| *TMPRSS2* | GCGGGGGGGA |  | 22.49% | 23.29% | 21.81% |  | 1.00 (Reference) |  |
|  | ACGGGGAGGG |  | 2.65% | 2.47% | 2.85% |  | 0.90 (0.59-1.36) | 0.614 |
|  | ACGGGGGGGA |  | 1.19% | 1.08% | 1.28% |  | 0.66 (0.33-1.34) | 0.253 |
|  | GCAAGGAGAA |  | 8.47% | 8.61% | 8.45% |  | 0.94 (0.72-1.23) | 0.663 |
|  | GCAAGGATAA |  | 3.03% | 2.69% | 3.35% |  | 0.74 (0.46-1.18) | 0.211 |
|  | GCGAAGAGAA |  | 7.74% | 8.90% | 7.05% |  | 1.17 (0.88-1.55) | 0.274 |
|  | GCGAAGAGGA |  | 2.28% | 2.25% | 2.39% |  | 0.89 (0.54-1.44) | 0.625 |
|  | GCGAAGATAA |  | 3.47% | 3.53% | 3.36% |  | 0.91 (0.61-1.38) | 0.668 |
|  | GCGAAGATGA |  | 1.58% | 1.69% | 1.37% |  | 0.99 (0.55-1.76) | 0.961 |
|  | GCGGGAGGGA |  | 1.58% | 1.77% | 1.33% |  | 1.25 (0.72-2.18) | 0.432 |
|  | GCGGGGAGGA |  | 1.31% | 1.26% | 1.34% |  | 0.89 (0.47-1.69) | 0.717 |
|  | GCGGGGAGGG |  | 11.22% | 11.15% | 11.24% |  | 0.95 (0.73-1.22) | 0.667 |
|  | GCGGGGATGA |  | 1.52% | 1.81% | 1.32% |  | 1.19 (0.67-2.11) | 0.550 |
|  | GCGGGGATGG |  | 11.76% | 12.63% | 11.00% |  | 1.06 (0.85-1.33) | 0.594 |
|  | GCGGGGGTGA |  | 4.58% | 3.68% | 5.42% |  | 0.63 (0.41-0.96) | 0.031 |
|  | GTGAAGAGGA |  | 4.79% | 4.32% | 5.18% |  | 0.79 (0.56-1.11) | 0.171 |
|  | GTGAAGATGA |  | 1.61% | 1.87% | 1.39% |  | 1.23 (0.67-2.27) | 0.507 |
|  | GTGAAGGGGA |  | 1.04% | 0.55% | 1.49% |  | 0.44 (0.19-1.00) | 0.051 |
| *ACE2* | GA |  | 54.27% | 55.12% | 53.57% |  | 1.00 (Reference) |  |
|  | AA |  | 36.61% | 34.87% | 38.01% |  | 0.93 (0.85-1.02) | 0.139 |
|  | AC |  | 9.12% | 10.01% | 8.42% |  | 1.06 (0.91-1.24) | 0.453 |

^a^Haplotypes observed with >1% frequency in pool.

^b^Adjusted for age and sex.

**Table S5. The mutation frequencies of *ACE2* and the co-factors in colorectal cancer tissues by TCGA database.**

| Gene | Mutation rate (%)^a^ | Nonsense mutation (%) | Missense mutation (%) | Frame shift del (%) | Splice site (%) |
| --- | --- | --- | --- | --- | --- |
| *ACE2* | 15/3.07 | - | 11/73.33 | 1/6.67 | 3/20.00 |
| *FURIN* | 16/3.27 | 1/6.25 | 12/75.00 | 2/12.50 | 1/6.25 |
| *TMPRSS2* | 6/1.23 | 1/16.67 | 5/83.33 | - | - |

^a^Mutation frequency is calculated by person-time.

**Table S6. Stratification analyses for the association between mutation frequencies of three genes and different subgroups.**

| Gene | Age | | | Sex | | | Site | | |
| --- | --- | --- | --- | --- | --- | --- | --- | --- | --- |
|  | EOCRC (n/%) | LOCRC (n/%) | *P* | Male (n/%) | Female (n/%) | *P* | Colon (n/%) | Rectum (n/%) | *P* |
| *ACE2* | 5/5.88 | 10/1.84 | **0.023** | 5/1.49 | 10/3.40 | 0.117 | 12/2.61 | 3/1.75 | 0.737 |
| *FURIN* | 2/2.35 | 14/2.57 | 1 | 8/2.39 | 8/2.72 | 0.791 | 14/3.05 | 2/1.17 | 0.294 |
| *TMPRSS2* | 1/1.18 | 5/0.92 | 0.583 | 4/1.19 | 2/0.68 | 0.802 | 4/0.87 | 2/1.17 | 1 |

EOCRC, early-onset colorectal cancer. LOCRC, late-onset colorectal cancer.

Mutation frequency is calculated by person-time.
